# Recanalisation theraphy for acute ischemic stroke in cancer patients

**DOI:** 10.1038/s41598-021-91257-5

**Published:** 2021-06-02

**Authors:** Giovanni Merlino, Carmelo Smeralda, Gian Luigi Gigli, Simone Lorenzut, Sara Pez, Andrea Surcinelli, Alessandro Marini, Valentina Maniaci, Mariarosaria Valente

**Affiliations:** 1 Stroke Unit and Clinical Neurology, Department of Neuroscience, Udine University Hospital, Piazzale S. Maria della Misericordia 15, 33100 Udine, Italy; 2 Clinical Neurology, Udine University Hospital, Udine, Italy; 3 DAME, University of Udine, Udine, Italy; 4 Oncology Unit, Monfalcone Hospital, Monfalcone, Italy

**Keywords:** Neurology, Oncology

## Abstract

To date, very few studies focused their attention on efficacy and safety of recanalisation therapy in acute ischemic stroke (AIS) patients with cancer, reporting conflicting results. We retrospectively analysed data from our database of consecutive patients admitted to the Udine University Hospital with AIS that were treated with recanalisation therapy, i.e. intravenous thrombolysis (IVT), mechanical thrombectomy (MT), and bridging therapy, from January 2015 to December 2019. We compared 3-month dependency, 3-month mortality, and symptomatic intracranial haemorrhage (SICH) occurrence of patients with active cancer (AC) and remote cancer (RC) with that of patients without cancer (WC) undergoing recanalisation therapy for AIS. Patients were followed up for 3 months. Among the 613 AIS patients included in the study, 79 patients (12.9%) had either AC (*n* = 46; 7.5%) or RC (*n* = 33; 5.4%). Although AC patients, when treated with IVT, had a significantly increased risk of 3-month mortality [odds ratio (OR) 6.97, 95% confidence interval (CI) 2.42–20.07, p = 0.001] than WC patients, stroke-related deaths did not differ between AC and WC patients (30% vs*.* 28.8%, p = 0.939). There were no significant differences between AC and WC patients, when treated with MT ± IVT, regarding 3-month dependency, 3-month mortality and SICH. Functional independence, mortality, and SICH were similar between RC and WC patients. In conclusion, recanalisation therapy might be used in AIS patients with nonmetastatic AC and with RC. Further studies are needed to explore the outcome of AIS patients with metastatic cancer undergoing recanalisation therapy.

## Introduction

Patients affected by cancer have an increased risk of acute ischemic stroke (AIS) due to comorbid vascular disease and hypercoagulability^1–5^. Atherosclerosis, nonbacterial thrombotic endocarditis, disseminated intravascular coagulation, tumor embolism, and cerebral venous thrombosis represent the most frequent causes of AIS in cancer patients^6^. In addition, concurrent cancer is able to impair short-term outcome in AIS patients^7^. On the basis of these considerations, it is fundamental to improve knowledge regarding recanalisation therapy of AIS patients affected by cancer. Recently, a retrospective analysis of a large nationwide sample of inpatient hospitalizations from 1998 to 2015 showed that cancer patients with AIS received intravenous thrombolysis (IVT) about two thirds as often as AIS patients without cancer. Differently, use of mechanical thrombectomy (MT) was similar between patients with and without cancer during the study period^8^.

To date, very few studies focused their attention on efficacy and safety of recanalisation therapy in AIS patients with cancer, reporting conflicting results^9–16^. In addition, it is unknown whether the outcome of AIS patients with remote cancer (RC) is more similar to that of patients with active cancer (AC) or without cancer (WC). Hypothesizing that AIS patients undergoing recanalisation therapy might have a different outcome on the basis of their cancer status, we decided to perform this study comparing AC and RC patients with those without malignancy.

## Methods

### Study participants

We retrospectively analysed data from our database of consecutive patients admitted to the Udine University Hospital with AIS that were treated with recanalisation therapy, i.e. IVT, and MT ± IVT, from January 2015 to December 2019. Patients were followed up for 3 months. Patients showing symptoms onset within 4.5 h received IVT (0.9 mg/kg over 1 h) in accordance with international guidelines^17,18^. IVT was performed right after the native CT-scan if intracerebral haemorrhage (ICH) had been ruled out. Patients were considered eligible for MT if the following inclusion criteria were fulfilled: (1) presence of large vessel occlusion (LVO) in the anterior (intracranial internal carotid artery, anterior cerebral artery, middle cerebral artery, and extracranial internal carotid artery *plus* middle cerebral artery, i.e. tandem occlusion) or posterior (intracranial vertebral artery, basilar artery, and posterior cerebral artery) circulation as revealed by CT angiography (CTA); (2) symptoms onset within 6 h; (3) Alberta stroke program early CT score (ASPECTS) > 6 on direct CT scan^19^. Alteplase administration was continued until the angiographic suite would be ready and during the endovascular procedure. Patients presenting beyond the time window for IVT or with major contraindications to IVT underwent direct MT^18^. All patients treated with recanalisation therapy were re-scanned approximately 24 h after treatment, or sooner if there was clinical deterioration.

Based on a previous study by Masrur et al.^9^, our patients were classified according to their cancer history into those with AC, RC, and WC. AC patients were those with any current or previous metastatic disease (except brain metastases), those undergoing current treatment for a malignancy or those who were offered treatment but refused it. Patients were also considered as affected by AC when initial diagnosis of malignancy was made during hospitalization after the onset of AIS. RC patients were those whose records indicated an inactive past history of malignancy with no history or evidence of metastatic disease, and who had completed any planned treatments. After the end of treatment, the period of 1 year was used for defining the absence of evidence of active cancer. Patients with end-stage malignancy, i.e. subjects with a life expectancy of less than 6-months or who received palliative care, and those with metastatic brain lesions are not treated with recanalisation therapy at our institution.

Written informed consent was obtained from all patients or their representatives. The study was approved by the local ethics committee, *Comitato Etico Unico Regionale* (Ref. No. CEUR-2020-Os-173). All experimental protocols were approved by the local ethics committee, *Comitato Etico Unico Regionale*. All research was performed in accordance with relevant guidelines/regulations.

### Data collection

The following variables were collected: age, sex, vascular risk factors, laboratory findings, admission systolic and diastolic blood pressure, admission heart rate and previous pharmacological treatment. In addition, early ischemic changes on native CT-scan within the middle cerebral artery were graded according to the ASPECTS^19^. Regarding vascular risk factors, we adopted the following definitions: (1) previous transient ischemic attack/stroke was defined if the patient had a history of ischemic (transient attack or stroke) or haemorrhagic cerebrovascular disease; (2) the presence of cardiovascular disease was based on the history of previous ischemic heart disease and/or revascularization treatment using percutaneous coronary intervention/coronary artery bypass grafting; (3) atrial fibrillation was defined if the patient had past medical history of atrial fibrillation that had been confirmed in medical records; (4) high blood pressure was defined as the history of hypertension and/or use of antihypertensive medication; (5) a history of diabetes mellitus that had been confirmed in medical records and/or use of insulin/oral hypoglycaemic agents were considered for defining diabetes; (6) a presence of hypercholesterolemia was based on the use of lipid-lowering medications; (7) information on active tobacco use was used for defining patient as a current smoker. For laboratory tests, venous blood samples were drawn at our Neurology Unit, within 24 h after hospitalization, during the morning hours (range 06.00–08:00) after an overnight fast (at least 12 h).

Regarding malignancy, we collected information on: (1) type of cancer; (2) metastasis status; (3) modality of treatment; (4) impact of cancer on patient’s daily living abilities by means of the Eastern Cooperative Oncology Group (ECOG) performance score. The ECOG score ranges from zero (“fully active”) through three (”capable of only limited self-care”) to five (“dead”)^20^. Based on previous studies^21,22^, we classified our AC patients into those with ECOG score 0–1 versus those with score ≥ 2. Information on underlying causes of death was collected.

### Clinical assessment

The Trial of ORG 10172 in Acute Stroke Treatment (TOAST) classification was used to determine AIS subtypes based on their etiology^23^. The National Institute of Health Stroke Scale (NIHSS) score was adopted for determining stroke severity at admission and at discharge. Patients showing an improvement of ≥ 8 points on the NIHSS from baseline or a NIHSS score of 0 or 1 at discharge were considered as affected by major neurological improvement. Functional outcome was assessed by means of the modified Rankin Scale (mRS) at admission, based on pre-stroke disability, and 3 months after stroke. The mRS score after discharge was recorded at the patients’ routine clinical visit or through telephone interview with patients or their immediate caregivers. Patients with a mRS score comprised between 3 and 5 were considered functionally dependent. The presence of intracranial haemorrhage (ICH), as a consequence of the recanalisation therapy, was defined as any parenchymal hematoma (PH) based on the European Cooperative Acute Stroke Study (ECASS) morphologic definitions (ECASS PH-1 or PH-2)^24^, whereas presence of symptomatic intracranial haemorrhage (SICH) was based on the ECASS-III protocol^25^. Regarding recanalisation therapy, we collected information on: (1) time from symptom onset to IVT or MT; (2) time from hospital arrival to alteplase administration (door-to-needle time); (3) time from hospital arrival to groin puncture (door-to-groin time); (3) MT procedure duration; (4) recanalisation rate, assessed at the end of MT using the thrombolysis in cerebral infarction (TICI) classification and defined as successful recanalisation when a TICI 2b-3 was achieved.

### Outcome measures

The following primary endpoints were analysed: (1) 3-month dependency; (2) 3-month all-cause mortality; (3) presence of SICH. Secondary endpoints were: (1) no major neurological improvement at discharge; (2) in-hospital all-cause mortality; (3) presence of ICH. All the outcome measures were collected as part of our routine clinical practice in patients affected by cerebrovascular events.

### Statistical analysis

Data are displayed in tables as mean and standard deviation, if not otherwise specified.

Differences across the different groups were assessed by means of the Chi square test for categorial variables. One-way analysis of variance for normally distributed continuous variables, and the Kruskal–Wallis test for non-normally distributed continuous variables and for ordinal variables were used. Post-hoc analysis was performed by means of the Bonferroni test or the Dunn test, when appropriate. The Kolmogorov–Smirnov test with Lilliefors significant correction was used to assess normal distribution of data.

Rates of primary and secondary endpoints were separately calculated for patients receiving IVT and for those undergoing MT ± IVT.

The impact of cancer status on primary outcome measures was evaluated by multiple logistic regression analysis with WC patients as reference category both in patients receiving IVT and in those undergoing MT ± IVT. Age, baseline NIHSS score, and pre-stroke mRS were included in the model as potential confounding variables. Systolic blood pressure was added to other confounders in the analysis that evaluated the association between cancer status and SICH occurrence.

All probability values are two-tailed. A p value < 0.05 was considered statistically significant. Statistical analysis was carried out using the SPSS Statistics, Version 22.0 (Chicago, IL, USA).

## Results

### Baseline characteristics

**Figure 1:**
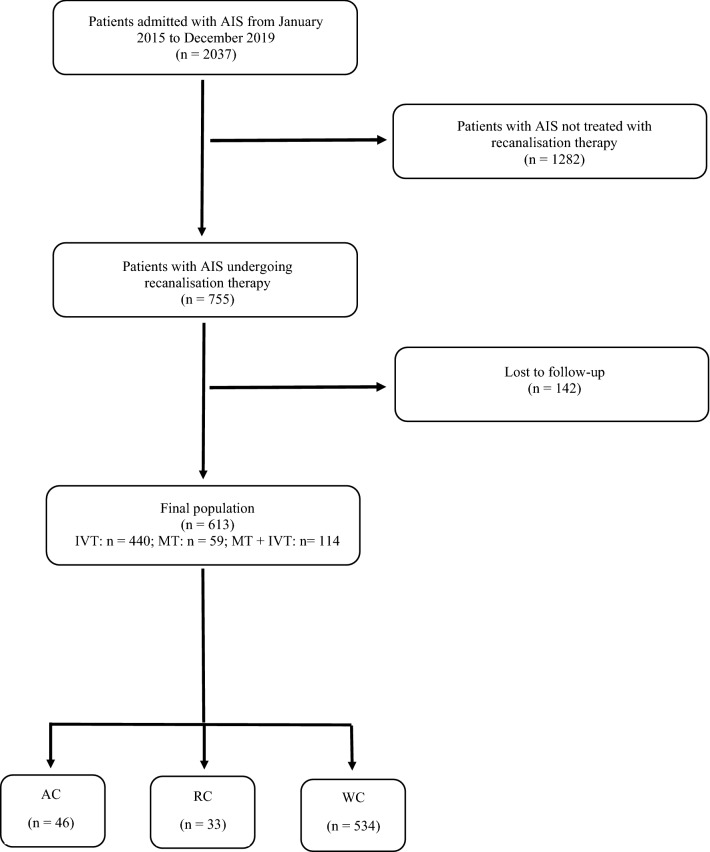
Flow diagram of the study. *AIS* acute ischemic stroke, *IVT* intravenous thrombolysis, *MT* mechanical thrombectomy, *AC* active cancer, *RC* remote cancer, *WC* without cancer.

During the study period 2037 patients were admitted for AIS, of them 755 were treated with recanalisation therapy. We lost to follow-up 142 patients (18.8%), thus the remaining 613 AIS patients were included in the study. Of them, 440 (71.8%) were treated with IVT, and 173 with MT, i.e. 114 (18.6%) received MT combined with alteplase, and 59 (9.6%) undergoing direct MT. Seventy-nine patients (12.9%) had either AC (*n* = 46; 7.5%) or RC (*n* = 33; 5.4%). These data are summarised in the flow diagram of the study (see Fig. 1). Clinical characteristics of AC patients are reported in Table 1. An ECOG score of 0–1 was observed in 43 (93.5%) AC patients. None among cancer patients presented AIS during a hospitalization for the malignancy. Types of cancer in RC patients were the following: prostate cancer in eight patients (24.2%); breast cancer in seven patients (21.2%); non-Hodgkin lymphoma in four patients (12.1%); endometrial cancer in three patients (9.1%); penile cancer in three patients (9.1%); kidney cancer in two patients (6.1%); laryngeal cancer in two patients (6.1%); urothelial cancer in two patients (6.1%); gastric cancer in one patient (3%); lung cancer in one patient (3%).

**Table 1:** Clinical characteristics of patients with active cancer.

Cancer type	No. (%) (n = 46)	Metastatic, No. (%) (n = 14)	Current cancer treatment, No. (%) (n = 14)
Breast	7 (15.2)	0	5/7 (71.4)
Colorectal	7 (15.2)	3/7 (42.9)	1/7 (14.3)
Prostate	7 (15.2)	1/7 (14.3)	5/7 (71.4)
Lung	6 (13)	3/6 (50)	0
Urothelial	6 (13)	1/6 (16.7)	1/6 (16.7)
Neuroendocrine	3 (6.5)	2/3 (66.7)	0
Kidney	2 (4.3)	1/2 (50)	1/2 (50)
Liver	2 (4.3)	0	0
Unknown primary site	2 (4.3)	2/2 (100)	0
Atrial myxoma	1 (2.2)	0	0
Endometrial	1 (2.2)	0	0
Multiple myeloma	1 (2.2)	N/A	0
Pancreas	1 (2.2)	1/1 (100)	1/1 (100)

The general characteristics of the three groups are presented in Table 2. Regarding baseline demographic and clinical characteristics, our groups did not show significant differences. In particular, age, sex, and vascular risk factors did not differ among the three groups. Apart from the protein level that was significantly lower in AC patients than in WC ones (p = 0.045, Bonferroni post-hoc test), the other laboratory findings were similar among groups. Although there was a trend for higher levels of C-reactive protein in AC patients, the prevalence of active infections was similar between the three groups (AC: 2.2%; RC: 3%; WC: 2.8%, p = 0.8). Causes of stroke, as classified by means of the TOAST, severity of neurological impairment at admission, as measured by the NIHSS score, and rate of pre-stroke functional independence, defined as an mRS ≤ 2, were not different among AC, RC, and WC patients. Although AC patients were treated more commonly with MT, alone or combined with IVT, than those in RC and WC groups, this difference was not statistically significant. Time from symptoms onset to recanalisation therapy, door-to-needle time, door-to-groin time, length of MT procedure, and successful recanalisation rate after MT were comparable among the three groups.

**Table 2:** General characteristics of the subjects according to the cancer status.

	AC (n = 46)	RC (n = 33)	WC (n = 534)	p
**Demographic data**				
Age, years	73.2 ± 11.2	74.4 ± 11.1	72.5 ± 13.1	0.665
Males, n (%)	25 (54.3)	19 (57.6)	289 (54.1)	0.928
**Vascular risk factors**				
Previous TIA/stroke, n (%)	6 (13)	1 (3)	53 (9.9)	0.321
Cardiovascular disease, n (%)	4 (8.7)	7 (21.2)	77 (14.4)	0.292
Atrial fibrillation, n (%)	9 (19.6)	8 (24.2)	97 (18.2)	0.674
Hypertension, n (%)	33 (71.7)	26 (78.8)	354 (66.3)	0.267
Diabetes mellitus, n (%)	7 (15.2)	6 (18.2)	93 (17.4)	0.922
Hypercholesterolemia, n (%)	15 (32.6)	14 (42.4)	133 (24.9)	0.053
Current smoking, n (%)	8 (17.4)	5 (15.2)	74 (13.9)	0.794
**Laboratory findings**				
Hb, g/dl	12.8 ± 1.8	13.5 ± 1.5	13.1 ± 1.7	0.151
Platelets, 10^3^/mm^3^	186.5 ± 65	186.8 ± 40.3	205.4 ± 60.2	0.052
aPTT ratio*	0.95 (0.88–1.04)	0.93 (0.86–1.07)	0.95 (0.87–1.08)	0.897
INR*	1.06 (1.01–1.11)	1.04 (1–1.16)	1.06 (1–1.14)	0.868
C-reactive protein, mg/l*	9.89 (2.57–14.35)	3.11 (1.28–9.04)	4.37 (1.95–11.68)	0.059
Protein, g/dl	6 ± 0.6	6.3 ± 0.4	6.2 ± 0.6	0.045
Albumin, g/dl	3.6 ± 0.4	3.6 ± 0.3	3.7 ± 0.4	0.312
Glucose, mg/dl*	124.5 (105.2–144.5)	115 (106–137)	126 (109–155)	0.181
eGFR, ml/min^a^	84.6 ± 20.9	84.1 ± 26.1	78.3 ± 24	0.111
Total cholesterol, mg/dl*	164 (138–190)	164 (139–193)	172 (146–200)	0.723
HDL cholesterol, mg/dl	51.5 ± 17.8	51.1 ± 14.6	52.9 ± 15.9	0.723
LDL cholesterol, mg/dl	94.9 ± 31.1	96.8 ± 34.8	100.3 ± 37.4	0.594
Triglycerides, mg/dl*	88 (70–131)	102 (79–135)	93 (67.5–126)	0.691
**Blood pressure and heart rate**				
Systolic blood pressure, mmHg	157.8 ± 22.8	163 ± 27	157.1 ± 24.9	0.447
Diastolic blood pressure, mmHg	89.2 ± 13.5	90.2 ± 14.7	86.6 ± 15.1	0.273
Heart rate, min	76.2 ± 15	77.4 ± 14.1	78.3 ± 15.7	0.716
**Antithrombotic treatment at admission**				0.646
Antiplatelets, n (%)	15 (32.6)	15 (45.5)	180 (33.7)	
Anticoagulants, n (%)	2 (4.3)	2 (6.1)	37 (6.9)	
Median baseline ASPECTS (range)	10 (8–10)	10 (8–10)	10 (7–10)	0.901
**Stroke subtypes based on TOAST classification**				0.692
Large arterial atherosclerosis, n (%)	5 (10.9)	7 (21.2)	83 (15.5)	
Cardioembolism, n (%)	21 (45.7)	14 (42.4)	209 (39.1)	
Small vessel occlusion, n (%)	3 (6.5)	4 (12.1)	48 (9)	
Other determined etiology, n (%)	0	0	14 (2.6)	
Undetermined etiology, n (%)	17 (37)	8 (24.2)	180 (33.7)	
**Baseline clinical characteristics**				
Median NIHSS score at admission (IQR)	12.5 (6–20)	9 (5–17)	9 (5–18)	0.128
Median NIHSS score at discharge (IQR)	3.5 (0.2–10)	1 (0–8.5)	2 (0–8)	0.318
Pre-stroke mRS 0–2, n (%)	43 (93.5)	31 (93.9)	480 (89.9)	0.566
**Type of recanalization therapy**				0.089
IVT, n (%)	24 (54.3)	25 (75.8)	390 (73)	
MT + IVT, n (%)	13 (28.3)	6 (18.2)	95 (17.8)	
MT, n (%)	8 (17.4)	2 (6.1)	49 (9.2)	
**Other information on recanalization therapy**				
Time from symptoms onset to IVT, min	172 ± 57.3	180.3 ± 59.9	164.5 ± 49.5	0.063
Time from symptoms onset to MT, min	191 ± 46.8	215 ± 37.2	213.5 ± 70.3	0.456
Door-to-needle time, min	76.2 ± 15	77.4 ± 14.1	78.3 ± 15.7	0.257
Door-to-groin time, min*	123.5 (105.7–143.2)	112.5 (85.5–137.2)	105 (80–135)	0.281
MT procedure length, min	79.9 ± 36.2	72.5 ± 35.5	77.1 ± 38.9	0.892
TICI 2b-3 after MT, n (%)	15 (71.4)	5 (62.5)	119 (82.6)	0.207

Data are presented as mean and standard deviation for normally distributed continuous variables. Non-normally distributed continuous variables are displayed as median and interquartile range and are identified by an asterisk (*).

*AC* active cancer, *RC* remote cancer, *WC* without cancer, *TIA* transient ischemic attack, *Hb* haemoglobin, *aPTT* activated partial thromboplastin time, *INR* international normalised ratio, *eGFR* estimated glomerular filtration rate, *HDL* high-density lipoprotein, *LDL* low-density lipoprotein, *ASPECTS* Alberta stroke program early CT score, *NIHSS* National Institute of Health stroke scale, *IQR* interquartile range, *mRS* modified Rankin scale, *IVT* intravenous thrombolysis, *MT* mechanical thrombectomy, *TICI* thrombolysis in cerebral infarction.

^a^eGFR was calculated using the Modification of Diet in Renal Disease formula.

### Association of cancer status with clinical outcomes among patients receiving IVT (univariate analysis)

**Figure 2:**
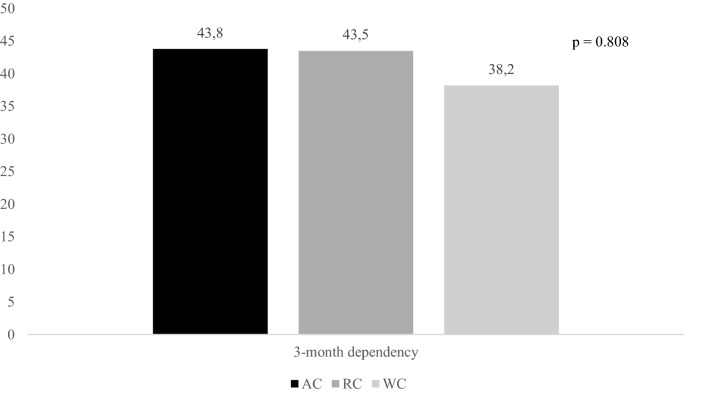
Rates of 3-month dependency according to cancer status among patients receiving IVT. *IVT* intravenous thrombolysis, *AC* active cancer, *RC* remote cancer, *WC* without cancer.

**Figure 3:**
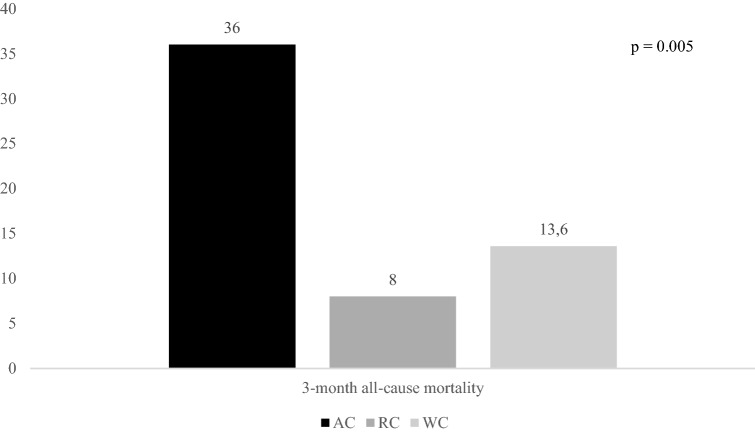
Rates of 3-month all-cause mortality according to cancer status among patients receiving IVT. *IVT* intravenous thrombolysis, *AC* active cancer, *RC* remote cancer, *WC* without cancer.

**Figure 4:**
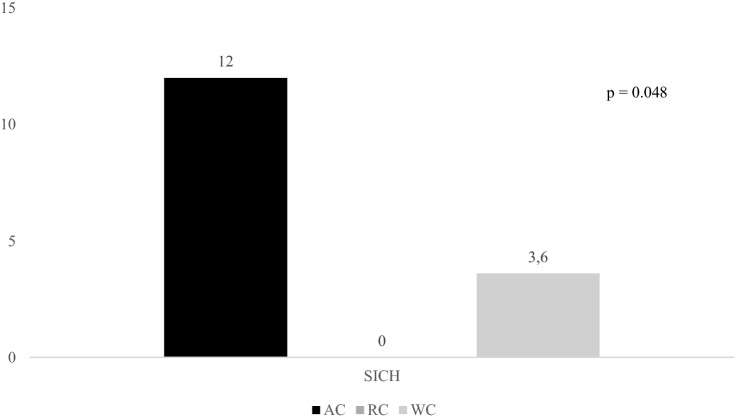
Rates of SICH according to cancer status among patients receiving IVT. *SICH* symptomatic intracranial haemorrhage, *IVT* intravenous thrombolysis, *AC* active cancer, *RC* remote cancer, *WC* without cancer.

Figures 2, 3, and 4 report rates of primary outcomes according to cancer status among patients receiving IVT. Prevalence of no major neurological improvement (48% for AC, 32% for RC, and 43.3% for WC, p = 0.471), in-hospital mortality (12% for AC, 8% for RC, and 5.6% for WC, p = 0.405), and ICH (20% for AC, 4% for RC, and 10.3% for WC, p = 0.168) was not statistically different among the three groups.

Although 3-month mortality was significantly increased in AC patients, we observed that stroke-related deaths were similar between these patients and WC ones (30% *vs.* 28.8%, p = 0.939). Causes of death, other than stroke, in AC patients were the following: sepsis (n = 3), and cancer progression (n = 3).

Whereas metastatic AC patients showed higher rates of 3-month mortality (55.6% *vs.* 14.6%, p = 0.017), functional dependency (50% *vs.* 42.9%, p = 0.593), and SICH (0% *vs.* 7.3%, p = 0.544) did not differ between patients with metastatic and non-metastatic AC.

### Association of cancer status with clinical outcomes among patients undergoing MT ± IVT (univariate analysis)

**Figure 5:**
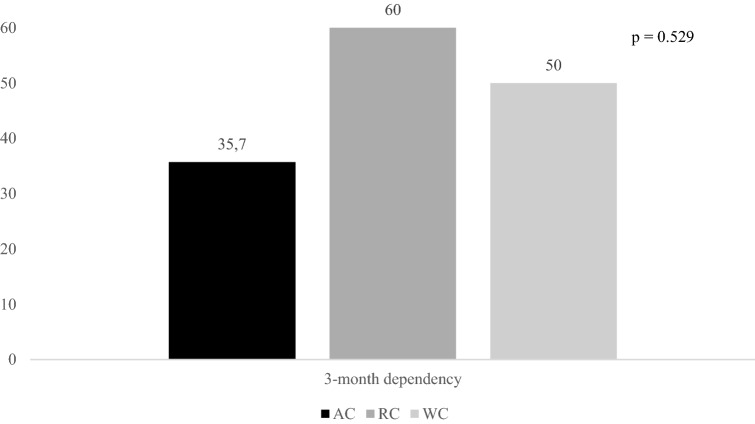
Rates of 3-month dependency according to cancer status among patients undergoing MT ± IVT. *MT* mechanical thrombectomy, *IVT* intravenous thrombolysis, *AC* active cancer, *RC* remote cancer, *WC* without cancer.

**Figure 6:**
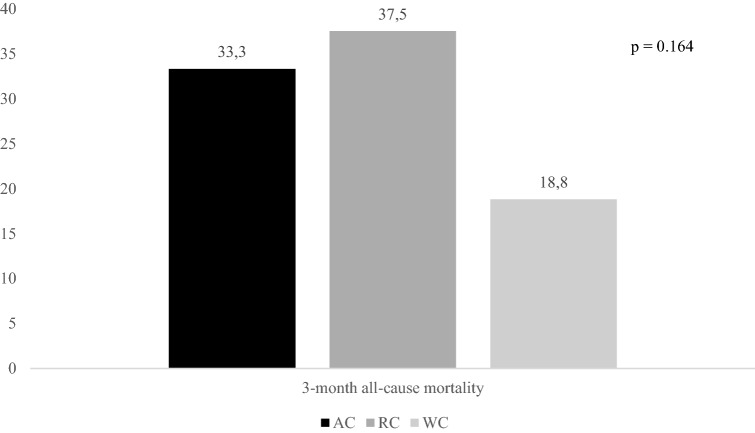
Rates of 3-month all-cause mortality according to cancer status among patients undergoing MT ± IVT. *MT* mechanical thrombectomy, *IVT* intravenous thrombolysis, *AC* active cancer, *RC* remote cancer, *WC* without cancer.

**Figure 7:**
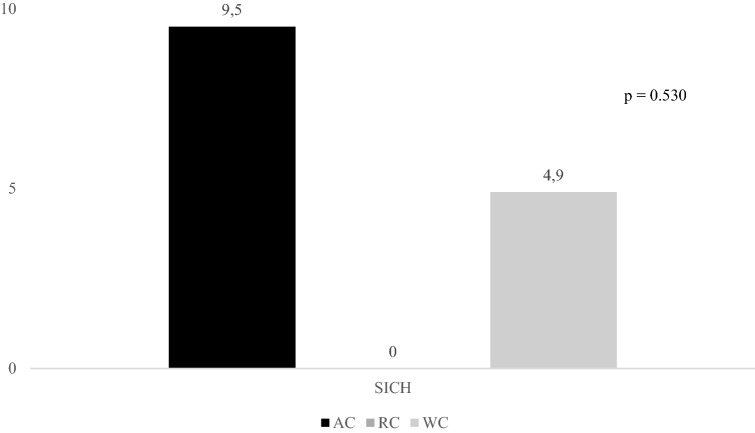
Rates of SICH according to cancer status among patients undergoing MT ± IVT. *SICH* symptomatic intracranial haemorrhage, *MT* mechanical thrombectomy, *IVT* intravenous thrombolysis, *AC* active cancer, *RC* remote cancer, *WC* without cancer.

Rates of primary outcomes according to cancer status among patients treated with MT ± IVT are reported in Figs. 5, 6, and 7. Whereas prevalence of no major neurological improvement (55% for AC, 62.5% for RC, and 36.6% for WC, p = 0.120) and in-hospital mortality (9.5% for AC, 25% for RC, and 10.4% for WC, p = 0.427) was similar, rates of ICH were statistically different among the three groups (52.4% for AC, 37.5% for RC, and 22.9% for WC, p = 0.014).

Among patients undergoing MT ± IVT, stroke-related deaths were significantly less common in AC subjects than in WC ones (14.3% vs. 69.6%, p = 0.015). Causes of death, other than stroke, in AC patients were the following: sepsis (n = 4), cancer progression (n = 1), and epigastric artery rupture (n = 1).

Prevalence of 3-month dependency (75% vs*.* 33.3%, p = 0.177), 3-month mortality (20% vs*.* 37.5%, p = 0.424), and SICH (20% vs*.* 4.2%, p = 0.320) did not differ between patients with metastatic and non-metastatic AC.

### Association of cancer status with clinical outcomes among patients receiving IVT (multivariate analysis)

As reported in Table 3, AC patients had a significantly higher risk of 3-month mortality than WC ones, even when multivariate analysis was performed. In contrast, the univariate association between the presence of AC and SICH occurrence was not confirmed after controlling for confounders. Independent predictors of the primary endpoints were the following: (1) age [odds ratio (OR) 1.04, 95% confidence interval (CI) 1.02–1.07, p = 0.001] and NIHSS score at admission (OR 1.15, 95% CI 1.10–1.20, p = 0.001) for 3-month dependency; (2) Age (OR 1.07, 95% CI 1.04–1.11, p = 0.001), and NIHSS score at admission (OR 1.15, 95% CI 1.10–1.20, p = 0.001) for 3-month all-cause mortality; (3) NIHSS score at admission (OR 1.04, 95% CI 1.02–1.11, p = 0.05) for SICH.

**Table 3:** Multivariate analysis of primary outcomes among AC, RC, and WC patients receiving IVT.

	WC Reference category	RC OR (95% CI)	AC OR (95% CI)
Three-month dependency^a^	1	1.69 (0.66–4.33) p = 0.276	1.64 (0.54–4.97) p = 0.381
Three-month all-cause mortality^a^	1	0.81 (0.16–4.08) p = 0.801	6.97 (2.42–20.07) p = 0.001
Presence of SICH^b^	1	0.97 (0.96–1.03) p = 0.501	3.66 (0.97–13.69) p = 0.054

*OR* odds ratios, *95% CI* 95% confidence intervals, *AC* active cancer, *RC* remote cancer, *WC* without cancer, *ICH* intracranial haemorrhage, *SICH* symptomatic intracranial haemorrhage.

^a^Adjusted for age, baseline NIHSS score, and pre-stroke mRS.

^b^Adjusted for age, baseline NIHSS score, pre-stroke mRS, and systolic blood pressure.

### Association of cancer status with clinical outcomes among patients undergoing MT ± IVT (multivariate analysis)

Odds of functional dependency, mortality, and SICH did not differ between AC and WC patients in multivariate analysis (see Table 4). Independent predictors of the primary endpoints were the following: (1) age (OR 1.04, 95% CI 1.01–1.07, p = 0.04) for 3-month dependency; (2) age (OR 1.06, 95% CI 1.01–1.11, p = 0.02), and pre-stroke mRS 0–2 (OR 0.15, 95% CI 0.03–0.68, p = 0.01) for 3-month all-cause mortality; (3) NIHSS score at admission (OR 1.10, 95% CI 1.07–1.25, p = 0.04) for SICH.

**Table 4:** Multivariate analysis of primary outcomes among AC, RC, and WC patients undergoing MT ± IVT.

	WC Reference category	RC OR (95% CI)	AC OR (95% CI)
Three-month dependency^a^	1	1.09 (0.17–7.11) p = 0.927	0.54 (0.17–1.75) p = 0.304
Three-month all-cause mortality^a^	1	1.65 (0.34–7.80) p = 0.535	2.36 (0.82–6.83) p = 0.112
Presence of SICH^b^	1	0.93 (0.89–1.12) p = 0.572	1.95 (0.33–11.63) p = 0.462

*OR* odds ratios, *95% CI* 95% confidence intervals, *AC* active cancer, *RC* remote cancer, *WC* without cancer, *ICH* intracranial haemorrhage, *SICH* symptomatic intracranial haemorrhage.

^a^Adjusted for age, baseline NIHSS score, and pre-stroke mRS.

^b^Adjusted for age, baseline NIHSS score, pre-stroke mRS, and systolic blood pressure.

## Discussion

This study demonstrated that AC patients affected by AIS did not have a worse outcome than WC subjects when treated with recanalisation treatment. Although rates of 3-month all-cause mortality were significantly higher in AC patients than in WC ones, when treated with IVT, we observed that stroke-related deaths were uncommon in AC subjects. Similarly, cancer status did not affect disability, mortality, and cerebral haemorrhagic complications in AIS patients undergoing MT ± IVT. In addition, clinical outcomes of RC and WC patients did not differ, regardless of treatment modalities for AIS.

To date, only few studies investigated the efficacy and safety of recanalisation therapy in AIS patients affected by cancer. In particular, data are very limited both for AC subjects and for RC ones. The largest investigations were performed by Murthy et al. and Weeda et al. who used the International Classification of Disease (ICD) codes for detecting cancer patients affected by AIS^10,13^. Although these studies included 32,576 and 13,993 patients, they suffered from major limitations: (1) information on stroke severity, stroke subtype, 90-day functional outcome, cancer staging, and modality of cancer treatment was lacking; (2) the use of administrative data for research depends largely on its accuracy and reliability, thus coding errors were a possibility.

Prevalence of 3-month dependency did not differ between AC and WC patients, regardless of treatment modalities for AIS. A previous Italian study by Sallustio et al. reported similar results^14^. Except for two researches conducted in Asian patients undergoing MT^12,15^, the remaining literature agreed that, in AIS patients undergoing recanalisation therapy, functional independence was not affected by the presence of concurrent malignancy^9,11,14,16^.

In 2011 Masrur et al. reviewed their database of AIS patients treated with urgent reperfusion treatment. Of 308 subjects included into the study, 18 and 26 were affected by active and remote malignancy, respectively. Patients with active malignancy had higher in-hospital mortality, but were more likely to die due to medical comorbidities instead of stroke-related causes^9^. More recently, prevalence of 3-month mortality has been reported more than double in patients with active malignancy with respect to controls in the case–control study of Sallustio et al. (29.1% vs*.* 12.5%). However, the most common causes of death were cancer-related and not stroke-related^14^. The above reported data are perfectly in line with our results. In fact, rates of 3-month mortality were higher in AC patients than in WC ones. However, stroke-related deaths were quite uncommon in patients with active malignancy, occurring in 30% of patients receiving IVT and in 14.6% of patients undergoing MT ± IVT. This lower stroke-related mortality was certainly not attributable to an earlier recognition and treatment of stroke in AC because of pre-existing hospitalization, since all AC patients were not hospitalized at the time of their stroke. Confirming this statement, rates of in-hospital mortality, that is in strong relationship with stroke consequences, were not different between AC and WC patients, regardless of treatment modalities for AIS. Why AC patients treated with MT ± IVT for AIS had lower rates of stroke-related deaths than WC ones remains unknown. Further studies should investigate this specific topic.

The risk of SICH after IVT and MT varies from 2 to 7%^26,27^. Coagulopathy and consequences of radiotherapy or chemotherapy may increase the odds of haemorrhage transformation after recanalisation therapy in AIS patients with active malignancy^28^. Data on SICH prevalence in AC patients show an extremely wide range from 0 to 16.2%^14,16^. This is because SICH definitions were different among studies. Only Sallustio et al. used our SICH definition based on the ECASS-III protocol. Whereas these authors did not report any SICH in both AC patients and controls, we observed a prevalence of 12% in patients with concurrent malignancy who received IVT and 9.5% in those who underwent MT ± IVT^14^. Methodological differences between the two studies might explain the discrepancy between our data and those of Sallustio et al.^14^. In our experience SICH occurrence did not differ between the three groups of AIS patients undergoing MT ± IVT, differently SICH was significantly more common in our AC patients, when treated with IVT, than in the other two groups. This univariate association was not confirmed when confounders, i.e. age, baseline NIHSS score, pre-stroke mRS, and admission systolic blood pressure, were included in the multivariate model. NIHSS at admission represented the only independent predictor of SICH in both IVT and MT ± IVT patients. Thus, it seems that AC patients treated with IVT have more haemorrhages because they have more severe stroke. These findings agreed with previous studies that did not report any significant association between the presence of active malignancy and the occurrence of haemorrhagic transformation after recanalisation therapy^9–11,14,16^.

A recent study by Yoo et al. compared 223 patients with nonactive cancer, 105 patients with active nonmetastatic cancer, and 140 patients with metastases. Since presence of metastases increased more than four times the risk of mortality, the authors suggested of using a careful approach when considering recanalisation therapy for AIS in patients with metastases. Similarly, we reported higher rates of 3-month mortality in patients with metastatic AC, but only when they were treated with IVT. In fact, we did not observe any significant difference regarding 3-month dependency, 3-month mortality, and SICH between patients with metastatic and non-metastatic AC when subjects underwent MT. These partial discrepancies might be due to the fact that, differently from our study, Yoo et al. enrolled patients not only treated, but also not treated with recanalisation therapy and used a longer follow-up period (6 months vs*.* 3 months)^29^. However, our results on metastatic patients should be considered with caution; in fact, these subgroup analyses were conducted on a small sample size having low statistical power.

Findings on efficacy and safety of recanalisation therapy in AIS patients with remote malignancy are very limited. Only Masrur et al. investigated this specific topic showing that RC patients had an unfavourable outcome. Rates of SICH were higher in RC patients than in AC and WC ones and in-hospital mortality did not differ between RC and AC patients^9^. Our patients with nonactive, remote malignancy receiving IVT had good clinical outcomes. Indeed, functional independence, mortality, and haemorrhagic transformation were similar between RC and WC patients. Although we did not observe any significant difference, RC patients undergoing MT showed higher rates of 3-month dependency, 3-month mortality, and in-hospital mortality than WC ones. We should underline that AIS patients enrolled by Masrur et al. received IVT within 3 h of stroke onset and were treated with intra-arterial thrombolysis when LVO occurred. Our data clearly support to use IVT in AIS patients with RC who should be considered as individuals with no previous history of malignancy. Additional data regarding MT in RC patients are needed.

Although the present study was one of the largest studies investigating outcome of cancer patients undergoing recanalisation therapy for AIS, nevertheless, it has some important limitations that merit mention. (1) This study has a significant selection bias since only patients undergoing recanalisation therapy for AIS were retrospectively included. We cannot exclude that some cancer patients were not assigned for recanalisation due to their advanced malignant disease and consequently they were not included in the study; (2) this is an observational study performed in a single centre with a limited sample size, which could have resulted in insufficient statistical power to detect true differences between groups; in fact, a power analysis was not conducted before data collection; (3) even after controlling for known confounders, residual confounding from unobserved factors might have affected our results; (4) due to the retrospective nature of the study, new or recurrent unrecognized malignancies might have occurred in RC patients causing a misdiagnosis (RC instead of AC); (5) since some of the 3-month mRS scores were indirectly recorded through telephone interview with patients or their caregivers, it might be possible that post-discharge functional status was not captured as well as in-hospital one; (6) finally, differently from patients treated with MT, for whom recanalisation outcomes are given (measured as TICI result), we were unable to report data on recanalisation rate among patients receiving IVT alone.

In conclusion, recanalisation therapy might be used in AIS patients with cancer. In particular, IVT, and MT ± IVT, seem to be safe and effective methods of treatment in subjects with nonmetastatic, active cancer and with nonactive, remote cancer. Regarding metastatic patients, further studies with larger sample size are needed to explore the real outcome of AIS patients with metastatic cancer undergoing recanalisation therapy. Until then, an individualized approach considering life-expectancy is warranted in this subset of patients.

## Data Availability

The datasets used and analysed during the current study are available from the corresponding author on reasonable request.
